# Effects of salinity and temperature on *in vitro* cell cycle and proliferation of *Perkinsus marinus* from Brazil

**DOI:** 10.1017/S0031182015001602

**Published:** 2016-02-18

**Authors:** FERNANDO RAMOS QUEIROGA, LUIS FERNANDO MARQUES-SANTOS, ISAC ALMEIDA DE MEDEIROS, PATRÍCIA MIRELLA DA SILVA

**Affiliations:** 1 Laboratório de Imunologia e Patologia de Invertebrados, Departamento de Biologia Molecular, Centro de Ciências Exatas e da Natureza, Universidade Federal da Paraíba, Campus I, CEP 58051-900, João Pessoa, PB, Brazil; 2 Laboratório de Biologia Celular e do Desenvolvimento, Departamento de Biologia Molecular, Centro de Ciências Exatas e da Natureza, Universidade Federal da Paraíba, Campus I, CEP 58051–900, João Pessoa, PB, Brazil; 3 Laboratório de Farmacologia Cardiovascular, Departamento de Ciências Farmacêuticas, Centro de Ciências da Saúde, Universidade Federal da Paraíba, CEP 58051–900, João Pessoa, PB, Brazil

**Keywords:** *Perkinsus marinus*, schizogony, ROS, salinity, temperature

## Abstract

Field and *in vitro* studies have shown that high salinities and temperatures promote the proliferation and dissemination of *Perkinsus marinus* in several environments. In Brazil, the parasite infects native oysters *Crassostrea gasar* and *Crassostrea rhizophorae* in the Northeast (NE), where the temperature is high throughout the year. Despite the high prevalence of *Perkinsus* spp. infection in oysters from the NE of Brazil, no mortality events were reported by oyster farmers to date. The present study evaluated the effects of salinity (5, 20 and 35 psu) and temperature (15, 25 and 35 °C) on *in vitro* proliferation of *P. marinus* isolated from a host (*C. rhizophorae*) in Brazil, for a period of up to 15 days and after the return to the control conditions (22 days; recovery). Different cellular parameters (changes of cell phase's composition, cell density, viability and production of reactive oxygen species) were analysed using flow cytometry. The results indicate that the *P. marinus* isolate was sensitive to the extreme salinities and temperatures analysed. Only the highest temperature caused lasting cell damage under prolonged exposure, impairing *P. marinus* recovery, which is likely to be associated with oxidative stress. These findings will contribute to the understanding of the dynamics of perkinsiosis in tropical regions.

## INTRODUCTION

Protozoa of the genus *Perkinsus* are facultative intracellular parasites of marine molluscs, in particular bivalves (Choi and Park, 2010; Villalba *et al.* 2011). This genus is widely distributed worldwide and consists of seven species. Two of them, *Perkinsus marinus* and *Perkinsus olseni*, require mandatory reporting to the World Organization for Animal Health because they cause mass mortality in oysters and clams (OIE, 2015), leading to important economic losses. Therefore, there is increasing interest in conducting a variety of studies, among them those aimed at determining the environmental conditions that favour the proliferation of the parasite in the host and that can lead to death (Burreson and Ragone Calvo, 1996; Oliver *et al.* 1998; Cáceres-Martínez *et al.* 2012).

The development of *Perkinsus* spp. *in vitro* isolation and propagation techniques (Gauthier and Vasta, 1993; La Peyre *et al.* 1993; Mclaughlin and Faisal, 1998; Casas *et al.* 2002, 2008) enabled the performance of more specific studies addressing morphological, biochemical, physiological and genetic aspects of these protozoan parasites (Sunila *et al.* 2001; Chu *et al.* 2002; La Peyre *et al.* 2006; Lund *et al.* 2007; Ascenso *et al.* 2009). *Perkinsus* spp. cells are generally present at the trophozoite phase when cultured *in vitro*. Throughout their development, under controlled conditions, trophozoites increase in size and subsequently undergo schizogony, which is characterized by successive internal cell divisions, with the formation of smaller cells that will generate new trophozoites (La Peyre *et al.* 1993; Sunila *et al.* 2001; Casas *et al.* 2008). Some isolates present zoosporulation under these conditions; however, this event is often restricted to a small percentage of the cell population (Dungan *et al.* 2007; da Silva *et al.* 2013).

The data collected so far in field studies (Burreson and Ragone Calvo, 1996; Oliver *et al.* 1998; Gullian-Klanian *et al.* 2008) and in *in vitro* assays (Burreson *et al.* 1994; La Peyre *et al.* 2006, 2010) lead to a consensus that high temperatures and salinities favour *P. marinus* proliferation, survival and infectivity. The simultaneous occurrence of these conditions may further contribute to the negative effects of the infection and can lead to the host's death, as traditionally observed for its typical host, the American oyster *Crassostrea virginica* (Smolowitz, 2013).

From the physiological point of view, little is known about the influence of temperature and salinity on *Perkinsus* spp. cells. In addition to cell proliferation and viability, other *in vitro* cellular parameters evaluated under these environmental conditions include metabolic activity (La Peyre *et al.* 2006), fatty acid synthesis (Lund *et al.* 2004), metabolism of lipids and lipase activity (Chu *et al.* 2003). In cells of some aquatic organisms and yeast (Lushchak, 2011; Zhang *et al.* 2015), including immune defence cells of bivalves (haemocytes, Hégaret *et al.* 2003; Chen *et al.* 2007), changes in temperature and salinity may alter the production of reactive oxygen species (ROS). Although these reactive molecules may be harmful to the cell itself at high concentrations, in some types of cells, they may act as secondary messengers in important physiological processes, such as cell differentiation and proliferation (see review by Sauer *et al.* 2001).

In Brazil, to date, *P. marinus* has only been reported in the Northeast (NE) region. The first case of *P. marinus* was reported in oysters of the species *Crassostrea rhizophorae* in the estuary of the Paraíba do Norte River (State of Paraíba; da Silva *et al.* 2013). Subsequently, this protozoan was detected infecting *Crassostrea gasar* oysters in the estuaries of the São Francisco River (State of Sergipe, da Silva *et al.* 2014) and the Mamanguape River (State of Paraíba, Queiroga *et al.* 2015). In these last two reports, *P. olseni* and *Perkinsus beihaiensis* species were also identified. In all of these studies, the prevalence of *Perkinsus* spp. was always high, reaching 100% in some cases.

Considering that high temperatures favour *P. marinus* infection and cause pathological consequences among *C. virginica* oysters and that in the Brazilian NE region high temperatures (mean maximum air temperature in Paraíba coast is 35 °C) predominate throughout the year, similar negative impacts might be expected among infected oysters in that region. However, in the NE of Brazil no mortality events were reported by oyster farmers or in the literature that could be associated with perkinsiosis. It is known though that when *C. gasar* oysters are infected by *Perkinsus* spp., the immune defence competence is reduced (i.e. numbers of haemocytes and proportions of their subtypes, phagocytic capacity and ROS production; Queiroga *et al.* 2013), which would contribute to the weakness of oysters making them more susceptible to opportunistic or pre-existing infections.

In the light of the above information, the present study aimed to evaluate the effects of salinity and temperature on the *in vitro* proliferation of *P. marinus* isolated from *C. rhizophorae* oysters in Brazil. For the first time, we analysed *P. marinus* cell viability and proliferation using parameters obtained by flow cytometry: cell density and morphology. It was possible to identify and to quantify the modifications of cell types that occurred under the effects of physical conditions. Moreover, ROS production was measured for the first time in *Perkinsus* sp. cells. Our results will help us in understanding the *P. marinus* infection dynamics (prevalence and intensity of infection) on the natural environment and the impact on potential hosts on the Brazilian coast.

## MATERIALS AND METHODS

### *Perkinsus marinus* isolate

The polyclonal isolate of *P. marinus* (CR-PB192) was obtained from one *C. rhizophorae* oyster sampled from the rhizophores of the red mangrove tree (*Rhizophora mangle*) from the estuary of the Paraíba do Norte River (06°58′16·6″S and 34°51′45·1″W), State of Paraíba, NE Brazil. This region has an annual average of 1800 mm rainfall and 26 °C of air temperature. At this estuary, seawater salinity and temperature range between 5–40 psu and 27–32 °C, respectively (Queiroga *et al.* 2012).

*P. marinus* trophozoites were isolated from infected gill fragments according to the protocol adapted from Casas *et al*. (2002). The isolate was established on 5th May 2014 and maintained in Dulbecco's modified Eagle's medium/Nutrient Mixture F-12 Ham (Sigma, Saint Louis, Missouri, USA; DME–HAM/F12, Gauthier and Vasta, 1995) at 20 psu and 25 °C. The isolate was identified as *P. marinus* by PCR (ribosomal RNA gene complexes, Casas *et al*. 2002) and the sequence obtained was submitted to GenBank (accession number KT692955).

### Effects of salinity and temperature on *P. marinus in vitro* proliferation

Two assays were performed independently to evaluate the effect of salinity and temperature on *P. marinus in vitro* proliferation. Prior to the assays, parasite cell suspension was held for 5 days of culture, and then rinsed by centrifugation (377 g for 10 min) and resuspended (10^6^ cells mL^−1^) in DME–HAM/F12 medium.

Salinity effects were assessed by propagating the isolate in three different media prepared at salinities: 5, 20 (control) and 35 psu. For each of the salinities, the cell suspensions were distributed (4 replicates) into 24-well plates and kept at 25 °C. Temperature effects were assessed by propagating the isolate in the medium at 20 psu. Similarly, cell suspensions were distributed (4 replicates) into three 24-well plates, which were maintained at different temperatures: 15, 25 (control) and 35 °C. These values of temperature and salinities were chosen in order to provide future comparison with the data of the natural environment of oysters.

*Perkinsus marinus* cell analyses were performed at different times: after 24 h, 48 h, 7 days and 15 days. In addition, an assay was conducted (*recovery assay*) to evaluate the proliferation capacity of *P. marinus* upon return to control conditions of salinity (20 psu) and temperature (25 °C). For this purpose, a sample of the cell suspension, from each replicate and treatment (all salinity and temperature conditions), from the 15th day of the experiment was used. The suspension was centrifuged (377 g for 10 min) and resuspended (1:3) in DME–HAM/F12 medium at 20 psu (the cell concentrations were not adjusted) and kept at 25 °C for more 7 days (total of 22 days).

Two samples were taken from each well (4 replicates and 6 treatments = 24 samples) at each time (24 h and 48 h, 7th, 15th and 22nd day = 120 samples) to perform analyses on flow cytometry and light microscopy.

### Flow cytometry analysis

The cell suspensions (4 replicates/treatment/time = 120 samples) were transferred to cytometry tubes and diluted in filtered sterile seawater with the same salinity used in the assays (1:1 until 48 h and 1:2 from the 7th day). The following parameters were analysed: cell morphology, density, viability and ROS production. Controls of unstained cells and dead cells (for viability assay) were used to adjust flow cytometer settings. The analyses were performed using a FACSCanto II flow cytometer (BD Biosciences, San Jose, California, USA). The samples were subjected to 30 s readings (cell density and viability) or 10 000 events (ROS production). The data obtained were analysed using Flowing software (Version 2.5·1), and the cytogram images were obtained using FlowJo software (Version X).

#### Cell morphology and density

Cell morphology was obtained from the cytograms with the FSC (Forward Scatter) and SSC (Side Scatter) detectors, which indicate cell size and internal cellular granularity, respectively. FSC *vs* SSC cytograms were also used to estimate the total number of *P. marinus* cells; considering the sample dilution (Hégaret *et al*. 2003). The proportion (%) of each *P. marinus* cell population was estimated.

#### Cell viability

Cell death was evaluated with the fluorophore propidium iodide (Sigma, Saint Louis, Missouri, USA; final concentration of 10 *µ*g mL^−1^), a double-stranded DNA intercalator incapable of crossing the plasma membranes of live cells. Unstained cells were also analysed. The percentage of cells with no fluorescence in the PE detector (564–606 nm) was determined to express cell viability (Soudant *et al.* 2005).

#### Production of ROS

The fluorophore 2′7′-dichlorofluorescein diacetate (DCFH-DA, Sigma Saint Louis, Missouri, USA; final concentration of 10 *µ*m) was used for ROS analysis. The hydrophobic molecule enters the cell and is subsequently cleaved by intracellular esterases. The oxidation caused by intracellular ROS converts DCFH into DCF, which emits fluorescence (Hégaret *et al.* 2003; Lambert *et al.* 2003). Reactive nitrogen species (RNS) may also oxidize DCFH (Wardman, 2007). Fluorescence was determined using the FITC detector (515–545 nm). Unstained cells were also analysed. ROS production was calculated by subtracting the geometric fluorescence mean of the labelled cells from the non-labelled cells.

### Light microscopy analysis

Considering that different cell phases (trophozoites, schizonts and zoospores) of *Perkinsus* spp. develop on *in vitro* culture (Sunila *et al.* 2001; Casas *et al.* 2002; Dang *et al.* 2015), light microscopy was used to correlate cell populations observed by this technique with those detected by flow cytometry.

The cell suspensions (4 replicates/treatment/time = 120 samples) were fixed in formaldehyde (final concentration of 2%) and used to identify and characterize (cell diameter and morphological description) each cell phase under a light microscope (Olympus BX45). Images of *P. marinus* cell cycle phases were taken with an Olympus Q-Color 5 digital camera.

### Statistical analysis

Two-way factor (i.e. temperature or salinity and time) analysis of variance (ANOVA) was used for all parameters (cell viability, cell density and ROS production), followed by the Bonferroni *post hoc* test. Significant difference was set at *P* < 0·01. All data are reported as mean ± s.e. Data analysis was performed using GraphPad Prism 5·0 software (San Diego, California, USA).

## RESULTS

### *Perkinsus marinus* phases *in vitro*

**Fig. 1.**
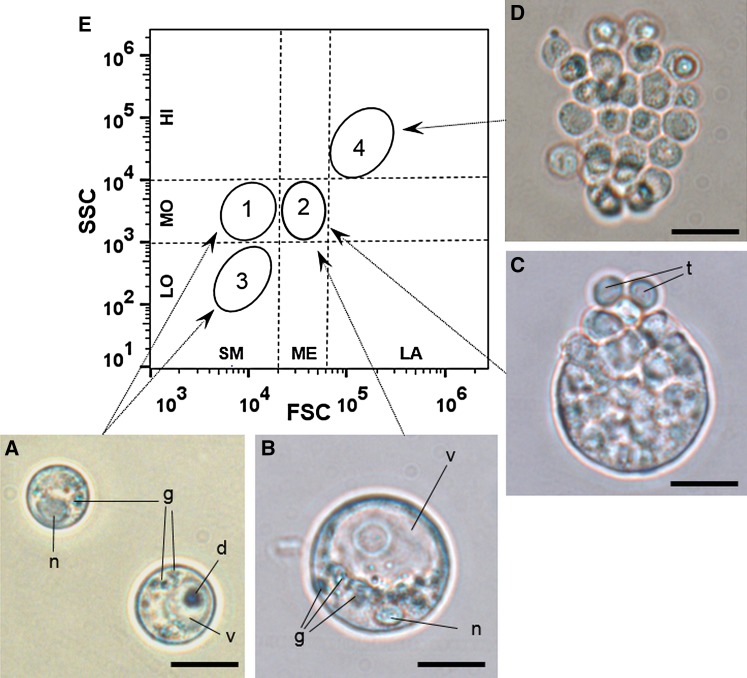
*Perkinsus marinus* cell types observed in *in vitro* cultures by light microscopy (pictures A – D) and depicted in flow citometry scatter plot (graphic E). (A) Small trophozoites. Note the peripheral nuclei (n), the large vacuole (v) containing a droplet (d) and the granules in the cytoplasm (g). (B) Large trophozoite. (C) Schizont releasing small trophozoites (t). (D) Cluster of sibling trophozoites. All micrograph bars = 10 *µ*m. (E) Representative flow cytometry scatter plot of *P. marinus* cell populations. Dashed lines show the limits of considered intervals for small, medium and large (SM, ME and LA, respectively) cell size (FSC – log scale) and low, moderate and high (LO, MO and HI, respectively) granularity (SSC – log scale). Circles show the positions of *Populations 1–4*. Arrows indicate possible associations between the cell types observed by light microscopy and populations detected by flow citometry.

By light microscopy, throughout the salinity and temperature assays, two cell types were observed (trophozoites and schizonts; Fig. 1A–D). Trophozoites (Fig. 1A and B) had diameters ranging from 5·3 to 32·1 *µ*m (11·8 ± 0·2 *µ*m) and were characterized by the presence of a prominent droplet inside the major cytoplasmic vacuole, which contained few granules. Schizonts had diameters ranging from 11·3 to 36·9 *µ*m (20·3 ± 0·3 *µ*m) and were round or oval cells with opaque cytoplasm. Schizonts releasing small trophozoites were observed (Fig. 1C). Moreover, small trophozoite aggregates (i.e. clusters of sibling trophozoites) were observed, with the main axes ranging from 19·2 to 66·1 *µ*m (37·9 ± 0·8 *µ*m) (Fig. 1D).

Flow cytometry morphological analyses (cell size *vs* granularity) indicated the presence of several cell populations, which are depicted in Fig. 1E and were numbered by the order they appeared in the cytograms. *Population 1* consisted of small-sized and moderate-granularity cells; *Population 2* consisted of medium-sized and moderate-granularity cells; *Population 3* consisted of small-sized and low-granularity cells; and *Population 4* consisted of large-sized and high-granularity cells.

Comparison of morphological characteristics of cell populations obtained with both methods (Fig. 1), indicates that: *Populations 1* and *3* would correspond to small trophozoites (Fig. 1A) with higher and lower internal granularities (respectively), *Population 2* would correspond to the large trophozoites (Fig. 1B) and schizonts (Fig. 1C), and *Population 4* would correspond to clusters of sibling trophozoites (Fig. 1D).

### Effect of salinity on *in vitro P. marinus* proliferation at 25 °C

Observation of cells under a light microscope showed the same patterns of occurrence of cell types at all salinities; trophozoites were observed at all-time points; however, the schizonts and clusters of sibling trophozoites appeared from the 7th and 15th day, respectively.

**Fig. 2.**
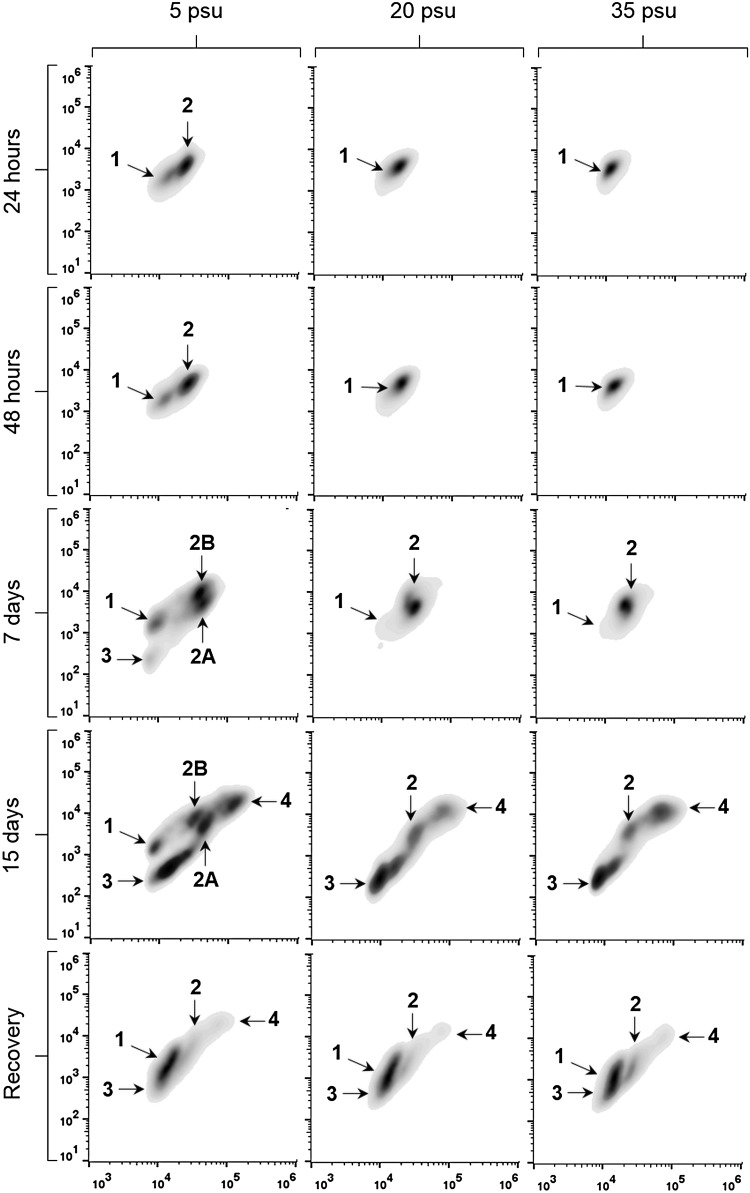
Density plot of *Perkinsus marinus* cells maintained at 5, 20 and 35 psu (columns) at different times (24, 48 h, 7 days, 15 days and in the recovery; rows). The *x*-axis indicates cell size (FSC), and the *y*-axis indicate cell granularity (SSC), both in log scale. Ten thousand events are represented at each cytogram. Numbers (1, 2, 2A, 2B, 3 and 4) indicate different cell populations.

The flow cytometry analysis indicated changes in the presence and proportions of the *P. marinus* cell populations over time as shown in Fig. 2 and Table 1. However, at the lowest salinity (5 psu), the changes were distinct from those occurring at higher salinities (20 and 35 psu). Until 48 h, at 20 and 35 psu, *P. marinus* presented exclusively as *Population 1*, whereas at 5 psu, *Population 2* emerged, corresponding to 70·5% (24 h) and 74·6% (48 h). Only on the 7th day, at 20 and 35 psu, did *Population 2* appear, 98·3 and 86·2%, respectively. In contrast, at 5 psu, *Population 2* subdivided into two distinct populations, *2A* (42·2%) and *2B* (39·1%); and *Population 3* also emerged (3%). On the 15th day, increased morphological variability was observed in all treatments, with all showing the simultaneous presence of the *Populations 2–4*. However, *Populations 1, 2A* and *2B* were sustained only at 5 psu (8·7, 11·8 and 21·6%, respectively). During the recovery period at 20 psu, cell morphology homogeneity was observed for all treatments; with *Populations 1–4*.

**Table 1.** Proportions (%) of *Perkinsus marinus* cell populations (Pop; 1, 2, 2A, 2B, 3 and 4) after culture at 5, 20 and 35 psu at different times (24h, 48 h, 7 days, 15 days and in the recovery). Data are reported as mean ± s.e. –  Absent population. Superscripted letters A and B refer to populations 2A and 2B, respectively.

Times	5 psu					20 psu				35 psu			
	Pop 1	Pop 2/2A/2B		Pop 3	Pop 4	Pop 1	Pop 2	Pop 3	Pop 4	Pop 1	Pop 2	Pop 3	Pop 4
24 h	29·5 ± 1·4	70·5 ± 1·3		–	–	100 ± 0	–	–	–	100 ± 0	–	–	–
48 h	25·4 ± 1	74·6 ± 1		–	–	100 ± 0	–	–	–	100 ± 0	–	–	–
7 days	16 ± 0·2	42·2 ± 4·9^A^	39·1 ± 2·9^B^	3 ± 1	–	1·8 ± 0·1	98·3 ± 0·1	–	–	13·8 ± 1·1	86·2 ± 1·1	–	–
15 days	8·7 ± 0·6	11·8 ± 0·6^A^	21·6 ± 1·2^B^	36·6 ± 2·9	21·3 ± 1·7	–	20·9 ± 0·2	66·4 ± 0·7	12·6 ± 0·6	–	27·1 ± 1·1	58·4 ± 1·8	14·5 ± 0·8
Recovery	74·9 ± 1·1	11·4 ± 1·1		9·8 ± 1·5	3·9 ± 0·5	55 ± 3·1	8·7 ± 0·8	33·5 ± 2·5	2·7 ± 0·3	45·1 ± 2·8	15·3 ± 0·4	36·5 ± 2·7	3·2 ± 0·3

**Fig. 3.**
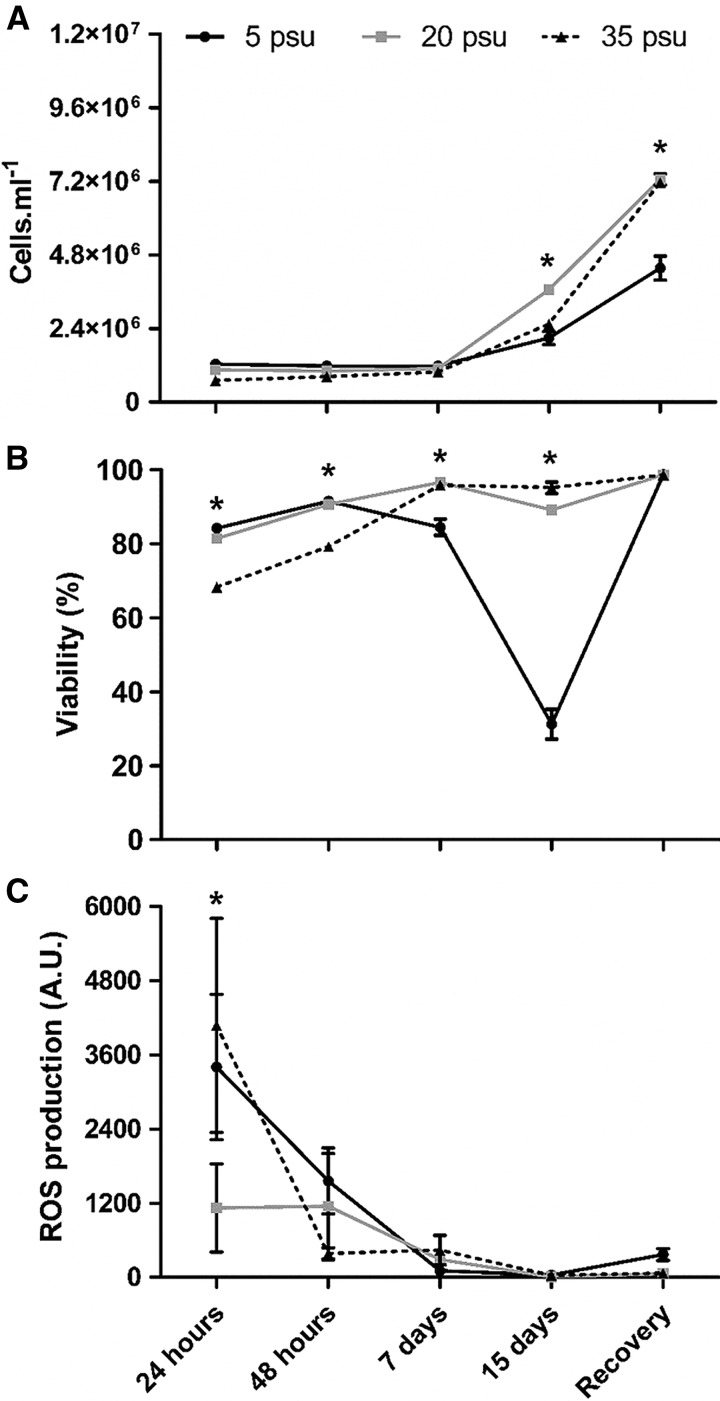
Cell density (A), cell viability (B) and ROS production (C) of *Perkinsus marinus* cells maintained at 5, 20 and 35 psu. Values are expressed as mean ± standard error. Asterisks indicate significant differences between treatments for each time point (*post hoc* Bonferroni test; *P* ⩽ 0·01).

Cell density, viability and ROS production of *P. marinus* cultured under different salinities are shown in Fig. 3. There was no cell proliferation at any salinity tested until the 7th day (Fig. 3A). On the 15th day, cell density at 20 psu was significantly higher (*P* < 0·001) (3·7 ± 0·10 × 10^6^ cells mL^−1^) than at 5 psu (2·1 ± 0·20 × 10^6^ cells mL^−1^) and at 35 psu (2·5 ± 0·10 × 10^6^ cells mL^−1^). However, in the recovery assay, the cell density previously at 35 psu (7·2 ± 0·04 × 10^6^ cells mL^−1^) was the same as that observed at 20 psu (7·3 ± 0·20 × 10^6^ cells mL^−1^) but was higher than that observed at the lowest salinity (4·4 ± 0·20 × 10^6^ cells mL^−1^; *P* < 0·001).

*Perkinsus marinus* cell viability at control salinity (20 psu) was maintained between 81·5 and 98·7% (91·3 ± 1·40%, Fig. 3B) throughout the study. For the other treatments, up to 48 h, only cells at the highest salinity (35 psu) presented reduced viability (79·2 ± 0·40%, *P* < 0·001). However, the cells maintained at 5 psu experienced slightly reduced viability on the 7th day (84·5 ± 2·20%, *P* < 0·001) and drastically reduced viability on the 15th day (31·3 ± 4·00%, *P* < 0·001). Curiously, the dead cells detected on the 7th day belonged mostly to *Population 2B* (83·2%), but not on the 15th day (28·8%). In all other cases, dead cells were evenly distributed in all populations. The cell viability at 35 psu (95·1 ± 1·50%) on the 7th and 15th day was equivalent to that of treatment at 20 psu. Recovery at 20 psu enabled cells previously at 5 psu to reach high viabilities (98·8 ± 0·10%) and values statistically equal to those of the other treatments.

ROS production (Fig. 3C) was higher for cells maintained at 5 and 35 psu only at 24 h (*P* < 0·01).

### Effects of temperature on *P. marinus* proliferation at 20 psu

By light microscopy, it was observed that cell culture kept at extreme temperatures analysed (15 and 35 °C) only showed trophozoites phase until the 15th day, but those kept at 25 °C also had schizonts and clusters of sibling trophozoites from the 7th day. However, recovery at 25 °C enabled the occurrence of all cell types for each tested temperature.

**Fig. 4.**
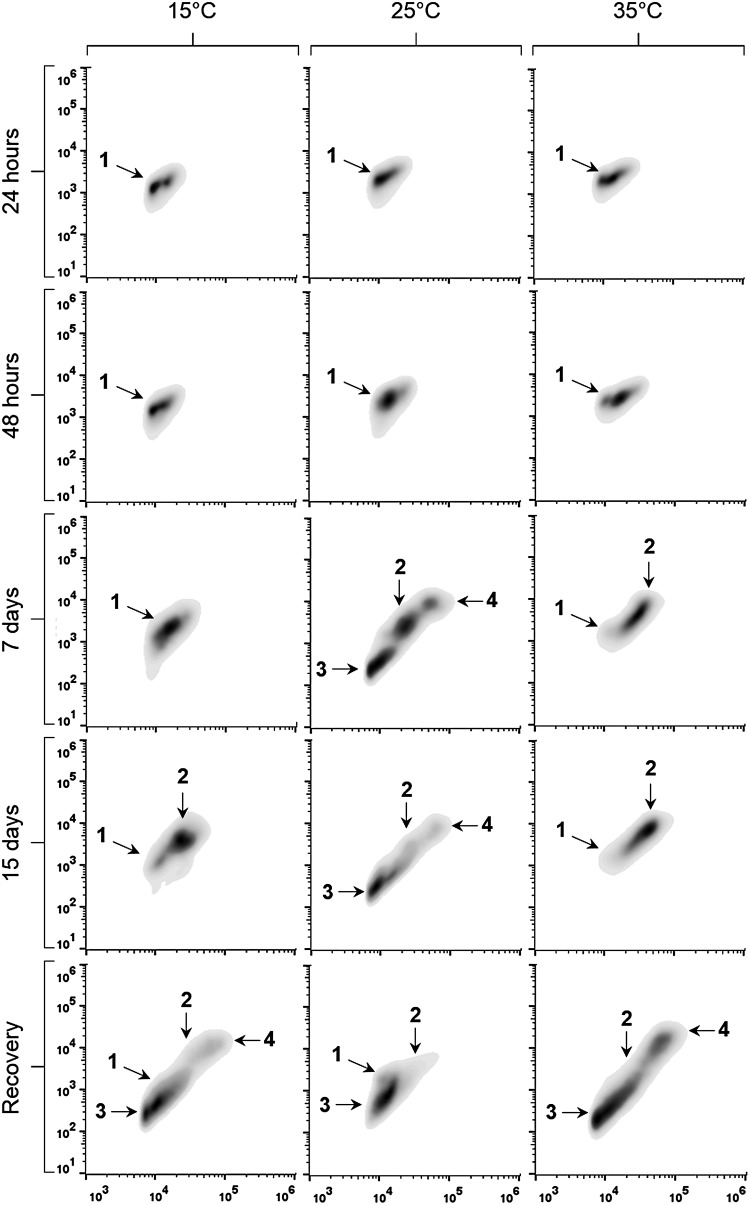
Density plot of *Perkinsus marinus* cells maintained at 15, 25 and 35 °C (columns) at different times (24, 48 h, 7 days, 15 days and in the recovery; rows). The *x*-axis indicates cell size (FSC), and the *y*-axis indicates cell granularity (SSC), both in log scale. Ten thousand events are represented at each cytogram. Numbers (1, 2, 3 and 4) indicate different cell populations.

Flow cytometry morphological analysis revealed changes in the presence and proportion of the *P. marinus* cell populations over time (Fig. 4 and Table 2). Notably, these modifications occurred slowly at extreme temperatures (15 and 35 °C). Up until 48 h, *P. marinus* cells presented exclusively as *Population 1* at all temperatures. On the 7th day, the cells maintained at low temperature (15 °C) sustained *Population 1*, whereas those kept at the highest temperature (35 °C) developed *Population 2* (83%), and those kept at 25 °C showed *Populations 2–4* (40·8, 39·3 and 19·9%, respectively). On the 15th day, *Population 2* emerged at the extreme temperatures (15 °C: 72·4%; 35 °C: 90·3%), together with *Population 1* (15 °C: 27·6%; 35 °C: 9·7%); whereas control temperature sustained *Populations 2–4*, although with different proportions (8·5, 83·6 and 7·9%, respectively). Recovery period at 25 °C induced cell changes for all treatments, although in different ways; the cells kept at 15 °C presented as *Populations 1–4*, those kept at 25 °C presented as *Populations 1–3*, while at 35 °C presented as *Populations 2–4*.

**Table 2.** Proportions (%) of *Perkinsus marinus* cell populations (Pop; 1, 2, 3 and 4) after culture at 15, 25 and 35 °C at different times (24 h, 48 h, 7 days, 15 days and in the recovery). Data are reported as mean ± s.e. –  Absent population.

Time	15 °C				25 °C				35 °C			
	Pop 1	Pop 2	Pop 3	Pop 4	Pop 1	Pop 2	Pop 3	Pop 4	Pop 1	Pop 2	Pop 3	Pop 4
24 h	100 ± 0	–	–	–	100 ± 0	–	–	–	100 ± 0	–	–	–
48 h	100 ± 0	–	–	–	100 ± 0	–	–	–	100 ± 0	–	–	–
7 days	100 ± 0	–	–	–	–	40·8 ± 3·6	39·3 ± 3·3	19·9 ± 1·8	17 ± 1·5	83 ± 1·5	–	–
15 days	27·6 ± 4	72·4 ± 4	–	–	–	8·5 ± 0·2	83·6 ± 0·7	7·9 ± 0·6	9·7 ± 1·6	90·3 ± 1·6	–	–
Recovery	6·4 ± 0·3	11·9 ± 0·2	73·3 ± 0·8	8·4 ± 0·4	17·4 ± 2·3	5 ± 0·2	77·62·2	–	–	20·6 ± 0·9	54·7 ± 3·6	24·7 ± 3·6

**Fig. 5.**
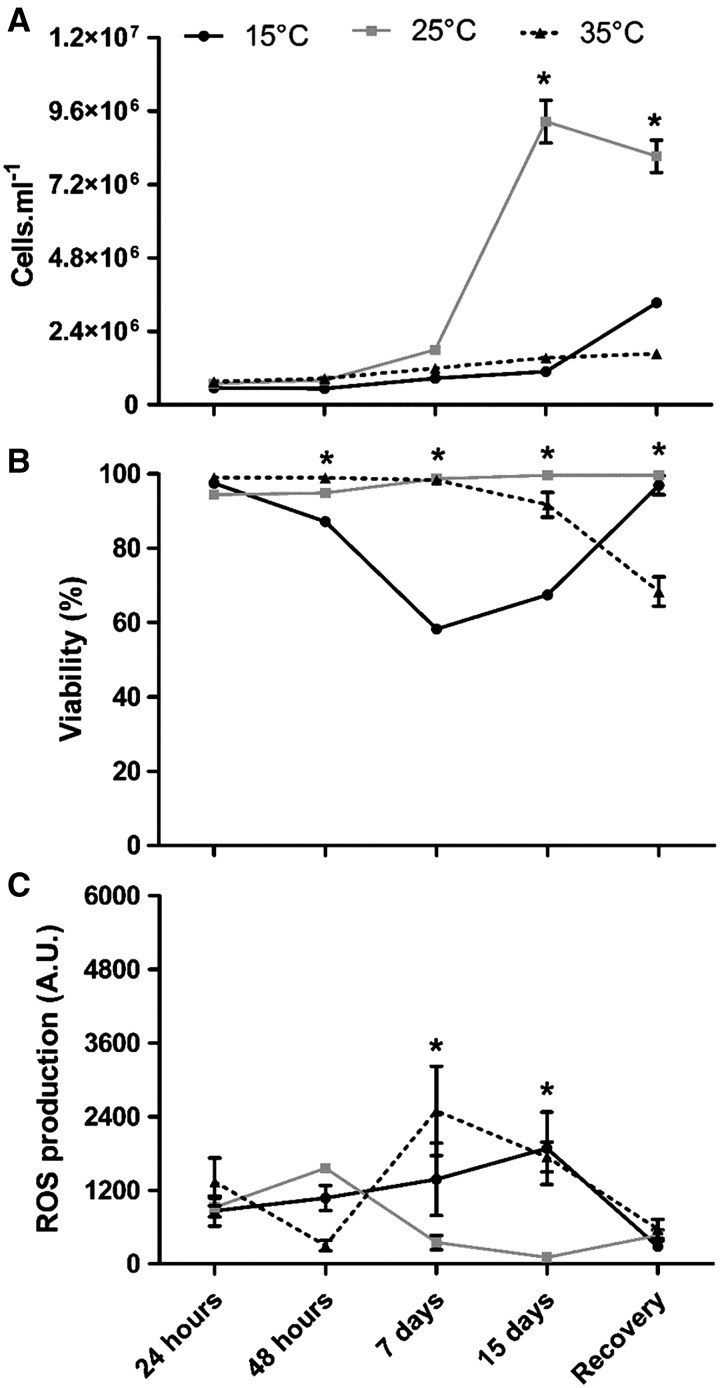
Cell density (A), cell viability (B) and ROS production (C) of *Perkinsus marinus* cells maintained at 15, 25 and 35 °C. Values are expressed as mean ± standard error. Asterisks indicate significant differences between treatments for each time point (*post hoc* Bonferroni test; *P* ⩽ 0·01).

Cell density, viability and ROS production of *P. marinus* cultured under different temperatures are shown in Fig. 5. *Perkinsus marinus* cell density did not differ among temperatures until the 7th day (Fig. 5A). After 15 days, the cells maintained at 25 °C reached a cell density higher (9·3 ± 0·70 × 10^6^ cells mL^−1^) than those of the other treatments (15 °C: 1·1 ± 0·01 × 10^6^ cells mL^−1^ and 35 °C: 1·53 ± 0·05 × 10^6^ cells mL^−1^; *P* < 0·001). Recovery at 25 °C did not modify the density of *P. marinus* cells kept at the highest temperature (35 °C: 1·7 ± 0·05 × 10^6^ cells mL^−1^), but induced their proliferation at the lowest temperature (15 °C: 3·3 ± 0·10 × 10^6^ cells mL^−1^). The density of cells kept at 25 °C (8·1 ± 0·50 × 10^6^ cells mL^−1^) was higher (*P* < 0·001) than to those of the other temperatures.

The cell viability of *P. marinus* cultured at 25 °C was high and constant throughout the experiment (97·4 ± 0·50%; Fig. 5B), but cells kept at a lower temperature (15 °C) exhibited an abrupt drop in viability after 48 h (87·1 ± 0·30%; *P* < 0·01), and further down after the 7th (58·3 ± 1·20%, *P* < 0·01) and 15th days (67·5 ± 0·80%) (*P* < 0·01). In contrast, cells kept at 35 °C showed a slight decrease in viability only on the 15th day (91·7 ± 3·30%; *P* < 0·01). In the recovery assay at 25 °C, the viability of cells kept at 15 °C (96·9 ± 2·50%) was equivalent to that of cells kept at 25 °C, whereas those maintained at 35 °C exhibited a significant reduction in viability (68·3 ± 3·90%; *P* < 0·001). In all conditions, dead cells were evenly distributed in all populations.

ROS production by *P. marinus* did not differ between the temperatures tested until 48 h (Fig. 5C). On the 7th day, cells kept at 35 °C presented ROS production higher than that of cells kept at 25 °C (*P* < 0·001), and those kept at 15 °C showed intermediate ROS production. On the 15th day, cells kept at 15 and 35 °C presented ROS production values higher than those of cells maintained at 25 °C (*P* < 0·01). In the recovery assay, ROS production was the same for all temperatures.

## DISCUSSION

The identification and *in vitro* propagation of *P. marinus* occurred for the first time in Brazil in 2013, derived from tissues of *C. rhizophorae* oysters from the estuary of the Paraíba do Norte River (NE, Brazil; da Silva *et al.* 2013). Since then, this protozoan parasite and others of the same genus (*P. olseni* and *P. beihaiensis*) have been identified as parasites of the *C. gasar* oyster (Queiroga *et al*. 2015; da Silva *et al.* 2014). However, *P. olseni* and *P. beihaiensis* have not yet been isolated and cultivated from Brazilian hosts.

Several *in vivo* (Oliver *et al.* 1998; Villalba *et al.* 2005; Gullian-Klanian *et al.* 2008) and *in vitro* (Burreson *et al.* 1994; Burreson and Ragone Calvo, 1996; La Peyre *et al.* 2006, 2010; Umeda *et al.* 2013) studies indicate that the protozoa of the genus *Perkinsus* are, in general, sensitive to extreme salinity and temperature conditions. The present study was the first to evaluate the effects of these two abiotic conditions on the physiology of *P. marinus* isolated from a Brazilian native host. The results indicate that the Brazilian *P. marinus* isolate is also sensitive to extreme temperature and salinity conditions, although in a more pronounced way than others isolates.

In the present study, trophozoites and schizonts, typical *Perkinsus* cells cultured *in vitro* (La Peyre *et al.* 1993; Ordás and Figueras, 1998; Sunila *et al.* 2001; Dang *et al.* 2015), were observed by microscopy. The development of one cell type into another was inferred from the morphological profile obtained by flow cytometry analysis and confirmed by microscopy. Here, cell density and morphological changes were analysed together by flow cytometry and used successfully to determine the *P. marinus* proliferation.

Among the cell types observed by microscopy, trophozoites showed three distinct morphological patterns (*Populations 1–3*) when observed on cytograms. *Populations 1* and *3* presented cells with the same size (the smallest cells observed), but different granularities. These cell populations may represent different trophozoite developmental phases (La Peyre *et al.* 1993; Sunila *et al.* 2001; Robledo *et al.* 2002; Casas *et al.* 2008). The difference in granularity may be associated with the capacity of *P. marinus* to store lipids in the form of cytoplasmic droplets (Chu *et al.* 2000, 2003). These lipids can be used for synthesis of structural components, mainly membranes, throughout its growth (Soudant and Chu, 2001; Chu *et al.* 2000, 2002; Lund *et al.* 2004). Lipid bodies accumulation also occurs with the intracellular parasite *Giardia lamblia* in culture (Stevens *et al.* 1997). Therefore, *Population 1* would include older trophozoites than *Population 3* because they would have accumulated more granules.

Over time, trophozoites increase in size (Fig. 1B) and undergo successive internal divisions, becoming schizonts (Fig. 1C), which contain immature trophozoites inside (Sunila *et al.* 2001; Casas *et al.* 2002; Dang *et al.* 2015). Large trophozoites and schizonts probably are contained in *Population 2* because of their similar size. When the new trophozoites grow, they eventually rupture the schizont's membrane and remain aggregated for some time (Ordás and Figueras, 1998; Sunila *et al.* 2001), as observed here under the light microscopy analysis, and suggested to be *Population 4* (flow cytometry). Therefore, clusters of sibling trophozoites were observed as individual events in the cytograms. Immature trophozoites will be released from the clusters to replenish *Population 3*. The simultaneous presence of *Populations 3* and *4* confirms the hypothesis of the cell cycle described above.

In the assay evaluating the effects of salinity and temperature, control conditions (20 psu and 25 °C) were the most favourable to *P. marinus* cell development, allowing sustained cell viability, cell phase changes and increase in density. In contrast, only at 5 psu, *Population 3* (small trophozoites) emerged on the 7th day, without clusters of sibling trophozoites development. This fact could be explained by an effect of low salinity inducing schizont rupture and the rapid separation of trophozoites from the cluster. Moreover, the subdivision of *Population 2* into *2A* and *2B* could not be simply explained by the high mortality observed in *Population 2B* (7th day), because cell death causes loss of membrane integrity, thus increasing cell size but not granularity, which as observed here. Instead, this fact corroborates the hypothesis discussed above; i.e. the presence of large trophozoites and schizonts in *Population 2* (*2A* and *2B*). Nevertheless, low salinity did not interfere with the progress of *P. marinus* cell phase changes, because the composition of cell populations was similar to that at the other salinities (15th and recovery).

The decrease of *P. marinus* proliferation at low salinities, as observed in this study, is a known phenomenon (La Peyre *et al.* 2006, 2010) associated with perkinsiosis infection dynamics (Oliver *et al.* 1998; Villalba *et al.* 2004; Gullian-Klanian *et al.* 2008; Soniat *et al.* 2012).

The low density of cells at 35 psu on the 15th day and the reduction in their viability until 48 h indicate that high salinity is also deleterious to the development of *P. marinus* from Brazil. Interestingly, at that time point, at both high and low salinity, the development of proliferative stages (schizonts and clusters of sibling trophozoites) was not affected. It is possible that extreme salinities caused a decrease in the proliferation rate, i.e. schizogony was not totally avoided but occurred at a slower pace and did not result in an increase in cell number.

Unlike salinity, there was no *P. marinus* proliferation at extreme temperatures (15 and 35 °C) until the 15th day. Other studies have observed that the optimal temperatures for the proliferation of *P. marinus* isolated from the American oyster *C. virginica* were between 15 and 35 °C (Dungan and Hamilton, 1995; Gauthier and Vasta, 1995). In the case of *P. olseni* isolated from *Ruditapes philippinarum* from Japan (Umeda *et al.* 2013), the optimal temperatures ranged between 20 and 34 °C. However, for an isolate of *Ruditapes decussatus* from Europe (Ordás and Figueras, 1998), the temperatures were lower (between 16 and 26 °C). The low proliferation of *P. marinus* when kept at 35 °C suggests that this isolate may be more sensitive to high temperatures than those mentioned above, but apparently equally as sensitive as the European *P. olseni* isolate.

The available studies focusing on *in vitro* proliferation of *Perkinsus* spp. contemplate only cell responses of the parasite under a single temperature and salinity condition (Dungan and Hamilton, 1995; Ordás and Figueras, 1998; La Peyre *et al.* 2006, 2010; Umeda *et al.* 2013). The present study also evaluated the response of *P. marinus* to its return to control temperature and salinity conditions. In this experiment, we aimed to evaluate the capacity of the parasite to recover from a potential stress situation. These assays could yield results closer to what is observed in the natural environment of oysters where the parasite is subjected to salinity and temperature fluctuations. Such fluctuations are typical of estuarine environments, where the tidal cycle and freshwater supply cause changes in the hydrological conditions.

In the recovery assessment, cells originally maintained at 5 and 35 psu and at 15 °C showed induction of proliferation accompanied by increased cell viability. These observations suggest that *P. marinus* has a strong capacity to recover after osmotic stress and a low temperature. Interestingly, recovery did not occur in the case of exposure to 35 °C. It is possible that the thermal stress suffered could have caused irreversible damage to the cells, contributing to their death. These observations could also explain why, despite the high prevalence of *P. marinus* among oysters in the NE region of Brazil, where high temperatures predominate, no mortality events have been reported to date (farmers, personal communication; da Silva *et al.* 2013, 2014; Queiroga *et al.* 2015). In this case, the high temperature in the NE region would be a limiting factor to the proliferation of the parasite.

The amount of ROS in the *P. marinus* cells was also affected in response to changes in salinity and temperature, although in a different way. This cell parameter has never been studied in protozoa belonging to this genus. What is known is how the parasite avoids the lytic molecules (ROS and RNS) produced and released by the host's defence cells (see review by Soudant *et al.* 2013).

In marine organisms, responses to increase in seawater temperature activate metabolic processes, resulting in increased ROS levels (Lushchak, 2011). Thus, this would explain the results observed in the present study when parasites were cultured at 35 °C. However, this mechanism does not explain the case of low temperature, which also induced ROS production. Lushchak (2011) speculates that in these rare cases, anti-oxidation mechanisms may be inhibited, although this possibility has not been confirmed. During the recovery period, *P. marinus* ROS values were equivalent to those under control conditions, supporting the direct association of this cell parameter with temperature. In marine animals, intracellular ROS production is also increased in cases of any transient changes (increases or decreases) in salinity. This increase derives from adaptations at the cell level, for example in the activity of proteins involved in energy metabolism and electrolytic balance (Lushchak, 2011). This phenomenon may have occurred in the case of *P. marinus* cells transitioning from a salinity of 20 psu to 5 or 35 psu, but for a short period (24 h).

The sustained elevation of ROS levels in *P. marinus* cells kept at 35 °C may have contributed to these cells’ inability to recover (as indicated by low cell proliferation and viability). Oxidative stress is a condition classically known to generate deleterious effects, such as lipid peroxidation (Mylonas and Kouretas, 1999), protein oxidation (Stadtman and Berlett, 1998) and even DNA oxidation (Cooke *et al.* 2003; Kelly *et al.* 2008). Nevertheless, the same does not apply to *P. marinus* cells kept at 15 °C because these specimens were able to recover even after being exposed to high ROS concentrations. It is probable that ROS do not cause the same effects at low temperatures, which could act as a protector.

In protozoa of the genus *Leishmania*, an increase in the ROS concentration is directly associated with the parasite's life cycle (Mittra and Andrews, 2013). A transient increase in the H_2_O_2_ concentration occurs before differentiation of the promastigote form into the amastigote form in cell culture. In this context, the ROS levels in *P. marinus* could also be associated with cell phase changes, since the sustained high ROS levels observed (15 and 35 °C) could have promoted the arrest in trophozoite phase (microscopy observation).

In temperate regions, infection by *Perkinsus* sp. follows temperature variations, and higher infection rates are reported in the warmer seasons and in higher salinity environments (Choi and Park, 2010; Villalba *et al.* 2011). The data provided in the present study suggest that the *P. marinus* isolated from the estuary of the Paraíba do Norte River is sensitive to both low and high temperatures and salinities. This estuary is located in the tropical region of Brazil, where the temperature varies little and the seasons are defined according to the rainfall (dry and wet seasons); rain in the autumn and winter (May–September) and dry conditions in the spring and summer (October–April). Thus, temperature would have less influence on the dynamics of infection by *P. marinus* in this region, but salinity would have a major role, causing temporal (through rainfall) and spatial (through localization up or down the river in the estuary) changes in perkinsiosis. Field studies in oyster populations in the NE region of Brazil focusing on *Perkinsus* spp. infection dynamics corroborate this hypothesis, such as low infection in the upper river and in the rainy season (Brandão *et al.* 2013; da Silva *et al.* 2014; Queiroga *et al.* 2015).

Based on the present study's data, we conclude that the *P. marinus* protozoan isolated from the *C. rhizophorae* oyster, an oyster native to the NE region of Brazil, presents *in vitro* higher sensitivity to extreme salinity and temperatures than other *P. marinus* isolates. Flow cytometry was an effective technique for studying physiological and proliferative (by cell morphological changes and density) aspects of the parasite. Our results will contribute to the understanding the dynamics of perkinsiosis in tropical regions and the impact of the *P. marinus* on hosts, which is of great relevance for predicting or preventing economic and environmental impacts.
